# Osteomyelitis in HIV pathogenesis, challenges, and therapeutic strategies

**DOI:** 10.1097/MS9.0000000000004326

**Published:** 2025-11-18

**Authors:** Emmanuel Ifeanyi Obeagu, O.G. Goryacheva

**Affiliations:** a Department of Biomedical and Laboratory Science, Africa University, Mutare, Zimbabwe; b Department of Polyclinic Therapy, Perm State Meical University named after Academician E.A. Wagner, Ministry of Health of the Russian Federation. 26 Petropavlovskaya St., Perm, Russian Federation

**Keywords:** bone infection, HIV, immunosuppression, inflammation, osteomyelitis

## Abstract

Osteomyelitis, a severe bone infection, presents a significant challenge in individuals living with HIV due to immunosuppression and an increased susceptibility to opportunistic pathogens. HIV infection leads to a weakened immune system, which, in conjunction with chronic inflammation and compromised bone health, increases the risk of developing osteomyelitis. This review delves into the complex pathogenesis of osteomyelitis in HIV patients, exploring the interplay between immune dysfunction, microbial invasion, and the subsequent bone damage that occurs. The diagnosis of osteomyelitis in HIV-infected individuals is often delayed due to nonspecific symptoms and the challenge of distinguishing it from other conditions. Imaging techniques, such as MRI and CT scans, are essential for identifying bone involvement, but microbiological confirmation can be challenging due to atypical pathogens. As a result, timely intervention becomes difficult and often leads to more severe infections and complications. This review highlights these diagnostic challenges and emphasizes the need for a more efficient and accurate approach to detection.

## Introduction

Osteomyelitis, a severe infection of the bone, is a significant clinical concern, particularly in immunocompromised individuals, including those living with HIV. It is typically caused by bacterial, fungal, or mycobacterial pathogens, leading to inflammation and bone destruction, and, if left untreated, can result in systemic infection and long-term disability. In HIV-infected individuals, the risk of developing osteomyelitis is increased due to immune suppression, altered inflammatory responses, and frequent exposure to opportunistic infections. The management of osteomyelitis in this population presents unique challenges, both in terms of diagnosis and treatment. The intersection of HIV-induced immunosuppression and the heightened risk of bone infections require a detailed understanding of the pathogenesis of osteomyelitis in these patients^[1,2]^. The pathogenesis of osteomyelitis in HIV patients is multifaceted, involving complex interactions between the host immune system, bone remodeling, and infectious agents. HIV-induced depletion of CD4+ T cells compromises the body’s ability to fight infections effectively, making HIV patients more susceptible to osteomyelitis. Studies have shown that the incidence of osteomyelitis is significantly higher in HIV-infected individuals, especially in those with advanced immunosuppression or uncontrolled HIV. For example, a study conducted by Pinto *et al* found that the incidence of osteomyelitis in HIV patients was approximately 12% higher compared to the general population, particularly among those with CD4 counts <200 cells/mm^[3]^. Furthermore, the prevalence of chronic osteomyelitis is often more pronounced in HIV patients, with a higher likelihood of recurrence and complications^[3,4]^. One of the key contributors to the development of osteomyelitis in HIV patients is chronic inflammation. HIV-induced immune activation leads to elevated levels of pro-inflammatory cytokines, which in turn promote osteoclast activation and bone resorption. This bone remodeling imbalance increases the risk of bone damage and infection. Research has shown that HIV patients exhibit significantly higher serum levels of inflammatory markers such as C-reactive protein (CRP), interleukin-6 (IL-6), and tumor necrosis factor-alpha (TNF-α), all of which have been linked to increased bone loss and higher susceptibility to infections like osteomyelitis. For instance, a study by Rodrigues *et al* (2017) found that HIV patients had significantly lower bone mineral density and higher levels of IL-6 and TNF-α compared to healthy controls, suggesting that inflammatory cytokine elevation plays a pivotal role in bone weakening and infection susceptibility^[5]^.

**HIGHLIGHTS**

- **Increased susceptibility**: HIV-induced immunosuppression heightens vulnerability to osteomyelitis, often caused by opportunistic pathogens.
- **Atypical pathogens**: Uncommon organisms, including mycobacteria and fungi, complicate diagnosis and treatment.
- **Diagnostic challenges**: Delayed diagnosis due to overlapping symptoms with other HIV-related bone disorders.
- **Therapeutic complexity**: Drug interactions between antiretroviral therapy and antibiotics pose treatment challenges.
- **Surgical considerations**: Advanced cases may require surgical intervention, complicated by poor wound healing in immunocompromised patients.

In addition to immune dysregulation, other risk factors specific to HIV patients contribute to the development of osteomyelitis. These include the use of intravenous drugs, which increases the likelihood of direct bacterial inoculation into bone tissue, and the presence of comorbidities such as tuberculosis (TB) and diabetes mellitus, both of which are more prevalent in the HIV population. TB, in particular, is a known cause of osteoarticular infections in HIV patients, with studies showing that approximately 10–15% of individuals with TB may develop osteoarticular involvement, including osteomyelitis. The immunosuppressive effects of HIV, combined with the immune-modulating impact of TB and diabetes, further exacerbate the risk of osteomyelitis in these patients^[6-8]^. Diagnosis of osteomyelitis in HIV patients remains challenging due to overlapping clinical symptoms with other opportunistic infections and inflammatory conditions. HIV patients with osteomyelitis often present with nonspecific symptoms such as fever, bone pain, and swelling, which can easily be mistaken for other conditions such as septic arthritis or osteoarthritis. Empirical studies have highlighted that HIV patients with osteomyelitis often have delayed diagnosis due to these nonspecific presentations. This delay in diagnosis can result in worsened outcomes, including increased risk of bone destruction and complications like septic arthritis or systemic infections^[9]^. Imaging plays a crucial role in the diagnosis of osteomyelitis, but it too presents challenges in HIV patients. While radiographs can reveal bone changes, such as periosteal reactions and bone destruction, more advanced imaging modalities like MRI and CT scans are often required for more accurate assessment. However, even these imaging techniques can sometimes be inconclusive, particularly in early stages of infection. A study by Silva *et al* noted that MRI scans in HIV patients with osteomyelitis often showed findings consistent with other HIV-related bone diseases, such as avascular necrosis or osteoporotic fractures. This overlap further complicates the diagnostic process and emphasizes the need for careful clinical correlation and microbiological confirmation^[10,11]^.

Microbiological confirmation of osteomyelitis is another major diagnostic challenge. Traditional culture methods, including blood cultures and bone biopsies, may fail to identify the causative pathogen in HIV patients due to the presence of opportunistic pathogens that are not routinely tested for in the general population. Studies by Venter *et al* have demonstrated that HIV patients with osteomyelitis are more likely to harbor atypical pathogens, such as *Mycobacterium tuberculosis, Candida* species, and other multidrug-resistant organisms^[12]^. This makes it essential to employ targeted diagnostic strategies and advanced culture techniques to identify the infecting pathogen, especially in HIV patients with risk factors for unusual infections^[13]^. Therapeutic management of osteomyelitis in HIV patients involves a combination of antimicrobial therapy, surgical intervention, and immune restoration through antiretroviral therapy (ART). Empirical antibiotic treatment often includes broad-spectrum antibiotics, while specific treatment is adjusted based on culture and susceptibility results. However, one of the biggest challenges in treating osteomyelitis in HIV patients is the high rate of antibiotic resistance, particularly in cases caused by *Staphylococcus aureus* and multidrug-resistant mycobacteria. Studies indicate that resistance to commonly used antibiotics, including penicillins and cephalosporins, is prevalent in HIV patients with osteomyelitis, necessitating the use of more potent or combination therapies. Moreover, ART plays a crucial role in improving immune function, which is essential for the resolution of infection and bone healing. Despite these advances, however, the management of osteomyelitis in HIV patients often requires a prolonged course of therapy and may include surgical intervention to remove necrotic bone tissue^[14]^.

## Aim

The aim of this review article is to explore the pathogenesis, diagnostic challenges, and therapeutic strategies for osteomyelitis in HIV-infected individuals.

## Methods

This narrative review was undertaken to provide a comprehensive synthesis of the current understanding of osteomyelitis in people living with HIV, with particular focus on pathogenesis, diagnostics, biomarkers, therapeutic strategies, and resource-adapted approaches. To identify relevant literature, a structured search was performed across multiple electronic databases, including PubMed, Scopus, Web of Science, and Google Scholar. The search strategy incorporated Medical Subject Headings (MeSH) and relevant keywords such as “HIV,” “osteomyelitis,” “bone infection,” “cytokines,” “biomarkers,” “molecular diagnostics,” “antimicrobial therapy,” “surgery,” and “resource-limited care,” combined using Boolean operators to ensure comprehensive coverage.

Articles published between 2000 and 2025 were considered, with priority given to studies published in the last 5 years to capture recent advances in molecular diagnostics, biomarker discovery, and therapeutic approaches. Inclusion criteria encompassed peer-reviewed original research, systematic reviews, meta-analyses, observational studies, clinical trials, and case series that reported on HIV-associated osteomyelitis in both adults and pediatric populations. Exclusion criteria included non-English publications, conference abstracts without full text, studies focused solely on non-HIV populations, and articles unrelated to bone infections.

Titles and abstracts were independently screened for relevance, followed by full-text evaluation of eligible studies. Key information was extracted, including mechanistic insights into bone resorption and immune dysregulation, cytokine pathways such as TNF-α, IL-17, and RANKL, diagnostic modalities ranging from traditional cultures to advanced molecular techniques [polymerase chain reaction (PCR), next-generation sequencing (NGS), and metagenomics], biomarker profiles including CRP, procalcitonin, osteocalcin, NT-proBNP, and point-of-care tools like lateral flow urine lipoarabinomannan assay (LF-LAM), as well as treatment approaches encompassing antimicrobial therapy, surgical interventions, and resource-adapted strategies such as task shifting, telemedicine, and generic antibiotic use. Discrepancies in data extraction were resolved through discussion and consensus among the authors. The collected evidence was synthesized qualitatively, with a focus on integrating mechanistic understanding, clinical applicability, and global relevance, rather than performing a quantitative meta-analysis. To enhance clarity and practical utility, visual elements including diagrams of pathogenic pathways, comparative tables of diagnostic modalities, and treatment algorithms were developed. While formal risk-of-bias scoring was not performed, studies were critically appraised for methodological rigor, sample size, and relevance, prioritizing primary studies and high-quality reviews to ensure contemporary and globally applicable insights.

## Pathogenesis of osteomyelitis in HIV

Osteomyelitis in individuals living with HIV arises from a complex interplay of immune dysregulation, microbial invasion, and bone remodeling disturbances. Beyond the general notion that “HIV weakens immunity,” mechanistic studies have elucidated specific cytokine-mediated pathways that directly contribute to bone susceptibility and destruction^[3,15]^. HIV infection induces chronic immune activation and progressive CD4+ T-cell depletion, which disrupts both innate and adaptive immune defenses. This immune dysregulation facilitates pathogen persistence and opportunistic infections, creating a permissive environment for osteomyelitis^[16,17]^. Among the key mediators, TNF-α plays a central role by stimulating osteoclast differentiation and activity, thereby promoting bone resorption. Concurrently, interleukin-17 (IL-17) – produced by Th17 cells – amplifies inflammation and recruits additional neutrophils and monocytes to infected bone, further enhancing local tissue damage. Receptor activator of nuclear factor kappa-B ligand (RANKL), expressed by osteoblasts and activated T cells, binds to its receptor RANK on osteoclast precursors, driving their maturation and bone-resorptive activity. The combined effects of TNF-α, IL-17, and RANKL establish a cytokine milieu that favors osteoclast-mediated bone destruction while impairing osteoblast-driven bone formation^[18,19]^.

In addition to host immune dysregulation, microbial factors significantly influence disease progression. Bacterial pathogens, such as *S. aureus* and *Salmonella* spp., frequently form biofilms, which confer resistance to immune clearance and antimicrobial therapy. In the context of HIV-induced immunosuppression, biofilm persistence is enhanced, leading to chronic infection and recurrent osteomyelitis. Recent studies highlight that biofilm-associated bacteria not only evade phagocytosis but also stimulate local pro-inflammatory cytokines, creating a self-perpetuating cycle of bone inflammation and degradation^[20]^.

## Diagnostic challenges of osteomyelitis in HIV-infected individuals

The diagnosis of osteomyelitis in HIV-infected patients presents significant challenges due to a combination of overlapping clinical manifestations, altered immune responses, and the diversity of causative pathogens. Accurate and timely diagnosis is critical to ensure appropriate treatment and prevent complications, but several factors complicate the identification of osteomyelitis in this immunocompromised population^[21]^.

### Non-specific clinical presentation

One of the primary challenges in diagnosing osteomyelitis in HIV-infected individuals is the nonspecific nature of its clinical presentation. In the early stages, osteomyelitis may present with mild symptoms, such as localized pain, fever, and swelling, which are also common in other HIV-related conditions like opportunistic infections, HIV-associated inflammatory disorders, or musculoskeletal manifestations of HIV. This makes it difficult for clinicians to distinguish between osteomyelitis and other differential diagnoses without further investigation. Additionally, HIV patients with advanced immunosuppression may not exhibit typical inflammatory responses to infections, which can obscure the clinical diagnosis. This reduced immune response often leads to more indolent or chronic presentations of osteomyelitis, further complicating the clinical evaluation^[22]^.

### Immunosuppressive effects of HIV

HIV infection, particularly in its advanced stages, is associated with immune dysfunction that impairs the body’s ability to mount a robust inflammatory response to infection. This immune suppression means that classic signs of osteomyelitis, such as localized warmth, redness, or swelling, may be absent or minimal. In some cases, fever, which is a hallmark of infection, may not be as prominent, further complicating the clinical picture. This diminished immune response can delay the recognition of osteomyelitis, as clinicians may not consider it a likely cause of symptoms in patients with HIV. Additionally, the presence of comorbid conditions, such as diabetes mellitus or TB, can further mask the presentation of osteomyelitis and contribute to diagnostic confusion^[23]^.

### Challenges in microbiological diagnosis

Microbiological diagnosis of osteomyelitis in HIV patients is challenging due to several factors, including the diversity of pathogens and the difficulty in obtaining adequate samples for culture. In immunocompromised individuals, osteomyelitis may be caused by a range of pathogens, including not only common bacteria like *S. aureus* but also opportunistic organisms such as *M. tuberculosis*, fungi, or atypical bacteria. Traditional culture methods may fail to identify less common pathogens, leading to misdiagnosis or delayed treatment. In some cases, cultures may yield negative results, even when osteomyelitis is present. This can occur due to low bacterial load, prior antibiotic treatment, or the presence of slow-growing organisms like mycobacteria or fungi, which are more difficult to culture. Furthermore, the presence of polymicrobial infections, which is more common in immunocompromised patients, complicates microbiological diagnosis and may lead to an underestimation of the diversity of infectious agents involved^[24]^.

### Radiological diagnostic limitations

Imaging studies, such as X-rays, CT scans, and MRI, are essential for the diagnosis of osteomyelitis, but they also present limitations in HIV patients. X-ray findings may not become evident until the infection has progressed to an advanced stage, such as bone destruction or sequestrum formation, which can delay early diagnosis. In early osteomyelitis, especially in cases caused by mycobacteria or fungi, radiological imaging may show minimal or nonspecific changes, which could lead to misinterpretation or missed diagnosis. MRI is the most sensitive imaging modality for detecting soft tissue involvement and bone marrow edema in osteomyelitis, but it may not differentiate between various types of infections, such as bacterial versus fungal or mycobacterial, without additional diagnostic tests. Moreover, in resource-limited settings, access to advanced imaging techniques like MRI may be restricted, further hindering early diagnosis and intervention^[25]^.

### Role of biopsy and histopathology

In cases where microbiological cultures are inconclusive or negative, biopsy and histopathological examination of bone tissue can be invaluable in confirming the diagnosis of osteomyelitis. However, obtaining a sufficient sample for biopsy in HIV patients can be challenging due to factors such as the site of infection, the presence of extensive soft tissue involvement, or the difficulty of performing invasive procedures in immunocompromised individuals. Additionally, biopsy results may not always yield definitive answers, especially when the infection is caused by slow-growing organisms, such as *M. tuberculosis* or fungal pathogens, which require specialized culture conditions. Furthermore, the histopathological findings in HIV patients with osteomyelitis can be nonspecific, with inflammatory cell infiltration and granuloma formation, which may overlap with other conditions like TB, fungal infections, or even HIV-associated bone lesions^[26]^.

### Co-infections and comorbidities

HIV-infected individuals often present with multiple co-infections or comorbidities, including TB, bacterial infections, and opportunistic fungal infections, which can complicate the diagnosis of osteomyelitis. For example, patients with HIV and TB may present with spinal osteomyelitis, which can be mistaken for other causes of back pain or musculoskeletal symptoms. The presence of co-infections can also lead to overlapping or masked clinical signs, as one infection may exacerbate the symptoms of another. This can lead to delays in diagnosing the underlying osteomyelitis or result in the misattribution of symptoms to other conditions. Additionally, HIV-related immunosuppression can result in the presence of multiple pathogens in the same infected site, making it more difficult to identify the specific organism responsible for the osteomyelitis^[27]^.

### Delayed diagnosis and disease progression

Given these diagnostic challenges, the timely identification of osteomyelitis in HIV patients is often delayed, which allows the infection to progress and complicates treatment. Delayed diagnosis can lead to the development of chronic osteomyelitis, which is harder to treat and may require prolonged antibiotic therapy, surgical debridement, or even amputation in severe cases. Chronic infections can also result in significant bone destruction, deformities, and functional impairments, leading to a poor quality of life for the patient. Furthermore, the prolonged inflammation and immune dysfunction associated with untreated osteomyelitis can lead to systemic complications, such as sepsis, which significantly increase the morbidity and mortality of HIV-infected individuals^[28]^.

## Therapeutic strategies for osteomyelitis in HIV-infected patients

The management of osteomyelitis in HIV-infected individuals is complex and requires an integrated approach that addresses both the underlying infection and the patient’s immunocompromised state. Effective treatment strategies must account for the diversity of pathogens involved, the potential for polymicrobial infections, and the altered immune response in HIV patients. A combination of antimicrobial therapy, surgical intervention, and HIV management is essential to optimize outcomes and reduce complications in this population (Table 1)^[29]^.

**Table 1:** Major therapeutic options in HIV-associated osteomyelitis, limitations, and outcomes

Therapeutic option	Description/use	Limitations	Reported outcomes
Broad-spectrum antibiotics	Empiric therapy targeting common bacterial pathogens (e.g., vancomycin and ceftriaxone)	Potential toxicity (renal and hepatic), risk of antimicrobial resistance, and drug–drug interactions with ART	Effective in acute infection; higher recurrence in immunocompromised patients without targeted de-escalation
Targeted antibiotics/de-escalation	Adjusted based on culture and sensitivity results	Requires reliable microbiological data; delayed initiation if cultures take time	Improved efficacy, reduced adverse effects, and lower resistance rates
Antimycobacterial therapy (TB co-infection)	Standard TB regimens for osteoarticular TB	Prolonged duration (6–12 months), potential hepatotoxicity, and drug interactions with ART	High cure rates when adherence maintained; delayed initiation can worsen bone destruction
Surgical debridement/abscess drainage	Removal of necrotic bone and drainage of abscesses	Invasive, requires surgical expertise, higher risk in advanced immunosuppression	Improved infection control and functional outcomes; recurrence less likely in well-selected patients
ART optimization/immune reconstitution	Initiation or adjustment of ART to improve immune function	Risk of IRIS, timing critical relative to infection	Enhances immune-mediated infection control; improves surgical and medical treatment outcomes
Adjunctive therapies (cytokine modulation/osteoclast inhibitors)	Emerging approaches targeting TNF-α, RANKL, or IL-17 pathways	Limited clinical data, experimental, not widely available	Preclinical studies show potential to reduce bone resorption; clinical efficacy still under investigation
Resource-adapted strategies (generic antibiotics, task-shifting, and telemedicine)	Cost-effective delivery of therapy, nurse-led monitoring, and remote consultations	Dependent on training, infrastructure, and local health care policies	Improves access and adherence in low-resource settings; enhances early detection and monitoring

### Antimicrobial therapy

The cornerstone of osteomyelitis treatment in HIV-infected individuals is antimicrobial therapy. However, the choice of antibiotics or antifungal agents must be guided by the causative organism, which can vary significantly in this population. Empirical broad-spectrum antibiotics, including *vancomycin* or *clindamycin*, are often initiated in the absence of microbiological results to cover for common pathogens like *S. aureus*, including methicillin-resistant strains (MRSA). Once the causative organism is identified, therapy should be tailored to the specific pathogen, and de-escalation to more targeted antibiotics or antifungal agents is recommended to minimize resistance and reduce unnecessary side effects. In HIV patients with osteomyelitis caused by opportunistic infections, such as *M. tuberculosis* or fungi, treatment regimens may require prolonged courses of therapy, sometimes lasting several months, and should be closely monitored for adverse reactions^[30]^. For cases of *S. aureus* osteomyelitis, particularly when caused by MRSA, long-term intravenous antibiotics may be required. In cases of osteomyelitis caused by mycobacteria, treatment with a combination of anti-TB medications, such as *rifampin, isoniazid*, and *pyrazinamide*, for 6–12 months is common. Antifungal therapy is indicated for fungal osteomyelitis, with drugs such as *fluconazole, amphotericin B*, or *itraconazole* depending on the specific pathogen. It is crucial to perform microbiological cultures and sensitivity testing to guide appropriate therapy and prevent the development of antibiotic resistance^[30]^.

### HIV management and immune reconstitution

Managing HIV itself is an integral component of osteomyelitis treatment in HIV-infected individuals. ART plays a crucial role in enhancing immune function, restoring CD4 T-cell counts, and controlling viral replication. In patients with advanced HIV or those who are immunocompromised, initiating or optimizing ART can improve immune responses, allowing the body to fight off infections more effectively. However, the timing of ART initiation in patients with active osteomyelitis must be carefully considered. In some cases, starting ART early in the course of infection may lead to immune reconstitution inflammatory syndrome (IRIS), a condition where the immune system responds aggressively to previously undiagnosed infections. Therefore, ART should be introduced carefully, with attention to the patient’s clinical condition and the risk of IRIS. Immune reconstitution with ART may also improve the efficacy of antimicrobial treatment by enhancing the body’s ability to fight infection. However, ART-related side effects, such as drug-drug interactions with antimicrobial agents, must be considered, and therapy should be adjusted accordingly to avoid toxicity and ensure optimal drug efficacy^[31]^.

### Surgical intervention

In some cases, surgical intervention may be necessary to manage osteomyelitis, particularly when there is extensive bone destruction, abscess formation, or sequestrum (dead bone) that cannot be adequately addressed with antimicrobial therapy alone. Surgical options include debridement of necrotic bone, drainage of abscesses, and sometimes bone grafting or orthopedic procedures to stabilize the affected area. Early surgical intervention can prevent the spread of infection to surrounding tissues and reduce the risk of chronic osteomyelitis. Surgical management is particularly critical in cases where osteomyelitis is caused by biofilm-forming organisms such as *S. aureus*, which are more difficult to treat with antibiotics alone. Biofilms protect bacteria from both immune responses and antimicrobial agents, necessitating the removal of infected tissue to achieve successful treatment. In addition, surgical debridement can reduce the burden of infection, improve healing, and restore function to the affected bone^[32]^.

### Pain management

Effective pain management is an essential part of treating osteomyelitis in HIV-infected individuals. Osteomyelitis is often associated with significant pain due to inflammation, bone destruction, and potential nerve involvement. Pain relief should be tailored to the severity of the pain and the patient’s overall health status. Nonsteroidal anti-inflammatory drugs NSAIDs, such as ibuprofen or naproxen, may be used for mild-to-moderate pain. For more severe pain, opioids may be prescribed on a short-term basis, with close monitoring to prevent misuse or dependence, especially in HIV patients who may be at higher risk due to polypharmacy and potential substance use disorders. In addition to pharmacological pain management, supportive measures such as rest, physical therapy, and the use of assistive devices can help alleviate discomfort and improve the patient’s quality of life. Pain management should be individualized, and regular reassessment is important to ensure that the patient’s needs are met throughout the course of treatment^[33]^.

### Adjunctive therapies

In certain cases, adjunctive therapies may be beneficial in the management of osteomyelitis in HIV-infected patients. These include hyperbaric oxygen therapy (HBOT), which has been shown to enhance the healing of bone tissue by improving oxygen delivery to infected areas and promoting angiogenesis. While the use of HBOT remains controversial in osteomyelitis, it may provide an additional therapeutic option, particularly in chronic or refractory cases. Nutritional support is also critical in the management of osteomyelitis in HIV-infected individuals. Malnutrition is common in this population and can impair immune function and wound healing. Ensuring adequate caloric intake, vitamin and mineral supplementation, and addressing any deficiencies is essential to support the body’s ability to combat infection and promote recovery. Protein supplementation, in particular, is important for tissue repair and immune function^[34]^.

### Monitoring and follow-up care

Continuous monitoring and follow-up are crucial in managing osteomyelitis in HIV-infected patients. Regular assessments of clinical response, laboratory tests (e.g., CRP and white blood cell count), and radiological imaging are necessary to evaluate the resolution of infection and identify any potential complications, such as the development of an abscess or sequestrum. Microbiological follow-up may also be required to monitor for recurrence or new infections. In patients undergoing prolonged antimicrobial therapy, regular checks for drug toxicity and side effects are important, as prolonged use of antibiotics or antifungal agents can lead to liver toxicity, renal dysfunction, or the development of resistance. Clinicians should also monitor for signs of drug-drug interactions between ART and antimicrobial agents, which may affect the efficacy of treatment and the patient’s overall health^[20]^.

### Multidisciplinary approach

Given the complexity of managing osteomyelitis in HIV-infected individuals, a multidisciplinary approach is essential. In addition to infectious disease specialists, orthopedic surgeons, and pain management experts, the care team should include HIV specialists, nutritionists, and rehabilitation therapists to address the diverse needs of the patient. This collaborative approach ensures comprehensive care and improves the likelihood of favorable outcomes. Furthermore, patient education plays a key role in treatment success. Educating patients about the importance of adhering to their antimicrobial regimen, attending follow-up appointments, and managing their HIV infection is essential for improving outcomes and reducing the risk of recurrence or complications^[35]^.

## Biomarkers in HIV-associated osteomyelitis

Biomarkers play a crucial role in the early detection, monitoring, and prognostication of osteomyelitis, particularly in immunocompromised populations such as people living with HIV^[36,37]^. Traditionally, markers such as CRP and procalcitonin have been used to assess systemic inflammation and bacterial infection. However, these markers are often nonspecific and may be influenced by concurrent infections, ART status, or chronic immune activation^[38,39].^ Recent advances have identified additional biomarkers that provide more nuanced insights into bone metabolism and cardiovascular involvement, both of which are relevant in HIV-associated osteomyelitis. Osteocalcin, a non-collagenous protein secreted by osteoblasts, serves as a marker of bone formation and remodeling. Decreased osteocalcin levels in HIV-infected patients may reflect impaired osteoblast activity and increased susceptibility to bone destruction. Conversely, NT-proBNP, a marker commonly associated with cardiac stress, has been observed to correlate with systemic inflammation and may indirectly reflect tissue damage and infection severity in advanced HIV^[40,41]^.

In regions with high burdens of TB, point-of-care diagnostic tools have become increasingly valuable. The LF-LAM allows rapid detection of active TB, including extrapulmonary involvement such as osteoarticular TB, which frequently complicates HIV-associated osteomyelitis. LF-LAM’s ease of use, rapid turnaround, and minimal infrastructure requirements make it particularly suitable for low-resource settings, where timely diagnosis can significantly influence outcomes^[42,43]^.

## Modern diagnostic approaches in HIV-associated osteomyelitis

Accurate and timely diagnosis of osteomyelitis in people living with HIV remains a challenge due to atypical presentations, immunosuppression, and the wide spectrum of causative pathogens. Traditional culture-based methods, while valuable, are often limited by low sensitivity, prolonged turnaround times, and difficulty in identifying fastidious or biofilm-forming organisms^[42]^. Molecular diagnostic techniques have therefore become increasingly important in contemporary practice. PCR allows rapid and specific detection of bacterial and mycobacterial DNA, even in culture-negative cases. NGS further enhances pathogen identification by providing comprehensive genomic profiles, enabling detection of mixed infections, antimicrobial resistance genes, and rare pathogens. Metagenomic approaches extend this capability by unbiased sequencing of all DNA/RNA in a clinical sample, offering unparalleled resolution for atypical or previously unrecognized organisms. These molecular tools are particularly valuable in HIV patients, where immunosuppression often complicates clinical presentation and conventional diagnostics may fail^[43,44]^.

To provide clarity on the practical use of these diagnostics, we have compiled a comparative table summarizing commonly used modalities in HIV-associated osteomyelitis. The table compares sensitivity, specificity, turnaround time, and approximate cost, highlighting the advantages and limitations of each method in various clinical settings, including resource-limited environments. Recognizing that advanced molecular tools may not be accessible in all regions, we also discuss ultrasound as a low-cost, point-of-care alternative. Ultrasound is particularly useful for detecting soft tissue abscesses, guiding percutaneous drainage, and monitoring treatment response. While less sensitive than MRI or CT for deep bone involvement, ultrasound offers a practical and accessible diagnostic option in rural and low-resource settings, complementing clinical assessment and laboratory testing^[45]^. By integrating molecular diagnostics with pragmatic imaging tools, clinicians can achieve more accurate, rapid, and context-appropriate identification of osteomyelitis pathogens in HIV-infected individuals, facilitating timely and targeted therapy (Table 2).

**Table 2:** Comparative overview of diagnostic modalities in HIV-associated osteomyelitis

Diagnostic modality	Sensitivity	Specificity	Turnaround time	Approx. cost	Comments/applicability
Culture (bone/aspirate)	Moderate (~50–70%)	High (~90%)	3–7 days	Low	Standard method; may miss fastidious or biofilm-associated pathogens
PCR (bacterial/mycobacterial DNA)	High (~85–95%)	High (~90–95%)	24–48 hours	Moderate	Rapid detection; requires lab infrastructure
Next-generation sequencing	Very High (~95–99%)	High (~95%)	48–72 hours	High	Detects mixed infections and antimicrobial resistance genes; limited access in low-resource settings
Metagenomics	Very High (~95–99%)	High (~95%)	48–72 hours	Very high	Comprehensive pathogen profiling; mostly research or tertiary centers
CRP/procalcitonin	Moderate (~60–75%)	Moderate (~70%)	Hours	Low	Useful for systemic inflammation monitoring; nonspecific
Osteocalcin/NT-proBNP	Variable (~60–80%)	Variable (~70–85%)	Hours	Moderate	Reflects bone turnover and systemic inflammation; adjunctive markers
LF-LAM (TB co-infection)	Moderate (~50–70%)	High (~90%)	<30 minutes	Low	Point-of-care; especially useful in TB-endemic, low-resource settings
Ultrasound	Low–Moderate (~50%)	Moderate (~70%)	Immediate	Low	Detects soft tissue abscesses; limited bone detail; suitable for rural/low-resource areas
MRI/CT	High (~90–95%)	High (~90–95%)	1–2 days	High	Gold-standard imaging for bone involvement; limited availability in resource-limited settings

## Antimicrobial therapy and treatment strategies in HIV-associated osteomyelitis

The management of osteomyelitis in HIV-infected individuals requires careful consideration of immune status, pathogen profile, and potential drug–drug interactions with ART. While broad-spectrum antibiotics, such as vancomycin or third-generation cephalosporins, are commonly employed empirically, HIV patients are at increased risk of adverse effects, prolonged infection, and multidrug-resistant pathogens, necessitating tailored antimicrobial strategies.

### Duration of therapy

The optimal duration of antimicrobial therapy in HIV-associated osteomyelitis is influenced by CD4 count, pathogen virulence, and bone involvement. In immunocompromised patients (CD4 < 200 cells/µL), therapy is generally extended compared to immunocompetent hosts to ensure adequate eradication. Typical courses range from 6 to 12 weeks for bacterial osteomyelitis, while mycobacterial infections often require 6–12 months of therapy, depending on drug susceptibility and treatment response. Monitoring clinical, radiological, and laboratory markers, including CRP, procalcitonin, and emerging biomarkers such as osteocalcin, helps guide therapy duration^[46]^.

### De-escalation strategies

De-escalation should be considered once culture and sensitivity results are available. This involves switching from broad-spectrum empiric therapy to targeted antibiotics with narrower coverage, minimizing toxicity and resistance selection. Regular monitoring for adverse drug reactions, especially nephrotoxicity or hepatotoxicity, is essential, given potential interactions with ART^[46]^.

### Integration with ART

Timing of ART initiation or modification is critical. Initiating ART during acute osteomyelitis can improve immune function but may precipitate IRIS, unmasking latent infections such as tuberculosis. Therefore, we recommend evaluating CD4 count and viral load, ruling out TB or other opportunistic infections, and coordinating ART with antimicrobial therapy to optimize immune recovery while minimizing complications.

### Surgical considerations and outcomes

Surgical intervention, including debridement, abscess drainage, and reconstruction, remains a cornerstone in cases of chronic or refractory osteomyelitis. Outcomes data from HIV-specific surgical cohorts indicate that patients with higher CD4 counts experience better postoperative healing and lower recurrence rates, whereas those with advanced immunosuppression may require staged procedures and prolonged postoperative antibiotic therapy. Multidisciplinary collaboration between infectious disease specialists, orthopedic surgeons, and HIV care providers is crucial to optimize outcomes^[47]^.

### Treatment algorithm

To streamline clinical decision-making, we have developed a treatment algorithm incorporating:

- CD4 count stratification (e.g., < 200 vs. > 200 cells/µL).
- Initial empiric therapy with broad-spectrum coverage.
- Early culture and sensitivity testing to guide de-escalation.
- TB rule-out using LF-LAM and radiology, particularly in endemic regions.
- Timing and coordination of ART to reduce IRIS risk.
- Surgical intervention in cases of chronic infection, abscess formation, or structural compromise.

This algorithm allows clinicians to deliver evidence-based, individualized care, balancing antimicrobial efficacy, immune status, and resource availability. By integrating pharmacologic, surgical, and ART considerations, it provides a comprehensive framework to improve outcomes in HIV-associated osteomyelitis.

## Resource-adapted strategies for HIV-associated osteomyelitis

Osteomyelitis in people living with HIV presents unique challenges in low-resource settings, where access to advanced imaging, molecular diagnostics, and specialized surgical care may be limited. To enhance clinical outcomes globally, resource-adapted strategies must balance efficacy, feasibility, and cost-effectiveness while ensuring equitable care.

### Use of generic antibiotics

In many low- and middle-income countries, access to brand-name antimicrobials is limited by cost and supply chain constraints. Generic antibiotics – including vancomycin, ceftriaxone, and amoxicillin-clavulanate – can provide effective empiric coverage when appropriately dosed and monitored. Evidence suggests that with correct stewardship and adherence, generic formulations offer comparable efficacy and safety to branded counterparts, supporting broader accessibility in resource-constrained environments^[48]^.

### Clinical scoring systems for diagnosis

Advanced imaging modalities, such as MRI or CT, are often unavailable in rural or underfunded health care settings. To overcome this, clinical scoring systems incorporating parameters such as localized pain, swelling, fever, laboratory markers (CRP and procalcitonin), and risk factors (CD4 count and prior infections) have been developed to guide diagnosis and treatment initiation. These pragmatic tools enable timely decision-making, reducing delays in therapy and preventing complications associated with late presentation^[49]^.

### Task-shifting and nurse-led models

Health care workforce shortages in many high-HIV-burden regions necessitate innovative care delivery models. Task-shifting, where nurses or mid-level providers assume roles traditionally performed by physicians, has proven effective in managing chronic infections. In osteomyelitis care, nurse-led models can coordinate antibiotic administration, monitor laboratory markers, provide wound care, and follow-up on treatment adherence. Training programs, clinical guidelines, and teleconsultation support ensure quality of care while expanding access in underserved areas^[48]^.

### Telemedicine for rural and remote populations

Telemedicine offers a powerful adjunct for rural health care delivery, enabling remote consultation with infectious disease specialists, orthopedic surgeons, and radiologists. Through video consultation, digital imaging transfer, and mobile health platforms, telemedicine facilitates timely diagnosis, therapeutic planning, and ongoing follow-up, reducing the need for travel and improving continuity of care. This approach is particularly beneficial in HIV-endemic regions, where patients face geographic and financial barriers to specialist care^[49]^.

### Advocacy for inclusive research

Despite the disproportionate burden of HIV and osteomyelitis in sub-Saharan Africa and other low-resource regions, most evidence originates from high-income countries. This review emphasizes the urgent need for inclusive research that reflects diverse patient populations, resource availability, and local epidemiology. Integrating global perspectives into clinical trials, observational studies, and implementation research will inform context-appropriate guidelines, optimize outcomes, and reduce inequities in osteomyelitis care among HIV-infected individuals worldwide^[50]^.

## Future perspectives and research directions

Despite advances in understanding and managing osteomyelitis in people living with HIV, significant gaps remain in both mechanistic knowledge and clinical practice. Future research must address these gaps to improve outcomes, particularly in regions with high HIV prevalence and limited health care resources^[36]^. Mechanistically, further studies are needed to elucidate the complex interplay between HIV-induced immune dysregulation, cytokine pathways such as TNF-α, IL-17, and RANKL, and osteoclast-mediated bone resorption. Understanding how chronic inflammation, microbial persistence, and biofilm formation contribute to recurrent or refractory osteomyelitis will inform the development of targeted therapies. Emerging interventions, including cytokine modulation, osteoclast inhibitors, and novel immunotherapeutics, hold promise but require rigorous preclinical and clinical evaluation in HIV-infected populations^[37,38]^.

From a diagnostic perspective, continued innovation in point-of-care and molecular technologies is critical. While PCR, NGS, and metagenomics have improved pathogen detection, their accessibility remains limited in low-resource settings. Research should focus on developing cost-effective, rapid, and field-adapted tools, such as portable PCR platforms or simplified metagenomic workflows, that can reliably detect bacterial, mycobacterial, and mixed infections. Integration of biomarkers like osteocalcin, NT-proBNP, and LF-LAM into clinical algorithms may further enhance early detection and individualized monitoring^[39,40]^. Therapeutically, there is a pressing need to generate high-quality evidence on optimal antimicrobial duration, de-escalation strategies, and ART integration in diverse HIV populations. Large, multicenter studies evaluating outcomes in patients stratified by CD4 count, viral load, and comorbidities are essential. Similarly, robust data on surgical interventions, including timing, technique, and postoperative management in immunocompromised hosts, are lacking, particularly in sub-Saharan Africa and other high-burden regions^[41,42]^.

The application of resource-adapted strategies warrants systematic investigation. Models such as task-shifting, nurse-led care, telemedicine, and the use of generic antibiotics have demonstrated feasibility but require evidence on long-term outcomes, cost-effectiveness, and scalability. Additionally, clinical scoring systems for diagnosis and monitoring should be validated across diverse settings to ensure broad applicability^[43–45]^. Future research must embrace a global, inclusive perspective, ensuring that findings reflect the epidemiology, health care infrastructure, and socioeconomic realities of regions most affected by HIV. Collaborative networks, capacity-building initiatives, and implementation science approaches are essential to translate mechanistic insights and innovative therapies into practical, sustainable interventions^[51]^.

## Recommendations

1. **Enhanced early diagnosis and screening:** Early and accurate diagnosis is crucial for the effective management of osteomyelitis in HIV-infected individuals. Improved diagnostic methods, including advanced imaging techniques and rapid molecular assays, should be developed and integrated into clinical practice to enable timely detection of osteomyelitis. Routine screening for bone infections in HIV-infected patients, particularly those with risk factors such as advanced immunosuppression, should be implemented to facilitate early intervention and prevent complications.
2. **Optimized antimicrobial stewardship:** Given the growing concern over antibiotic resistance in HIV-infected patients, a more targeted approach to antimicrobial therapy is essential. Health care providers should adopt an antimicrobial stewardship framework that ensures the appropriate use of antibiotics, minimizing overuse and preventing the development of resistance. Regular surveillance of local antimicrobial resistance patterns can help guide empirical treatment decisions and tailor therapies to individual patients, improving outcomes and reducing the risk of treatment failure.
3. **Integrated management of HIV and osteomyelitis:** A comprehensive, multidisciplinary approach to managing osteomyelitis in HIV patients is essential for achieving optimal outcomes. This includes coordination between HIV specialists, infectious disease experts, orthopedic surgeons, and other health care providers to deliver personalized care. Strengthening the integration of ART with the management of osteomyelitis is crucial to improve immune reconstitution and facilitate bone healing. Monitoring and optimizing ART regimens, particularly in cases of treatment failure or drug resistance, is vital for controlling HIV-related immunosuppression.
4. **Immune modulatory and adjuvant therapies:** Exploring immune-modulating therapies to enhance the immune response in HIV-infected patients with osteomyelitis could improve the body’s ability to fight infections and promote bone healing. Research into the use of immune checkpoint inhibitors, cytokine therapy, and other immunomodulatory agents should be prioritized to assess their potential in managing osteomyelitis. In addition, adjunctive therapies aimed at reducing inflammation and promoting tissue repair could complement conventional treatments, potentially improving the prognosis of affected individuals.
5. **Research on novel treatment modalities:** There is a critical need for the development of new and more effective treatment modalities for osteomyelitis in HIV-infected patients, especially given the increasing prevalence of multidrug-resistant pathogens. This includes the investigation of new antibiotics, antifungal agents, and alternative therapeutic strategies, such as bacteriophage therapy, antimicrobial peptides, and targeted drug delivery systems. Furthermore, research should focus on the exploration of personalized treatment plans that incorporate genetic, microbiological, and immunological factors to optimize outcomes.
6. **Improved access to care in low-resource settings:** Osteomyelitis remains a significant concern in resource-limited settings, where access to timely diagnosis, advanced imaging, and specialized treatments may be restricted. Public health efforts should focus on improving health care infrastructure, training health care workers, and increasing access to essential diagnostic tools and treatment options. Expanding access to ART, as well as creating awareness about the potential risk of bone infections in HIV-infected individuals, will help reduce the incidence and impact of osteomyelitis in these populations.
7. **Longitudinal studies and data collection:** Longitudinal studies and data collection are necessary to better understand the long-term effects of osteomyelitis in HIV-infected individuals. These studies should focus on the relationship between osteomyelitis and HIV progression, as well as the impact of various treatment regimens on bone health. Tracking patient outcomes over time will help to identify potential complications, improve clinical decision-making, and inform the development of guidelines for the management of osteomyelitis in HIV-infected patients.
8. **Public health initiatives and education:** Public health initiatives should raise awareness about the risks of osteomyelitis among HIV-infected individuals and encourage regular check-ups to detect early signs of bone infection. Educational campaigns should target both health care providers and patient

## Conclusion

Osteomyelitis in people living with HIV represents a complex interplay of immune dysregulation, chronic inflammation, and microbial persistence, posing unique diagnostic and therapeutic challenges. Advances in mechanistic understanding, including the roles of cytokines such as TNF-α, IL-17, and RANKL in bone resorption, have highlighted potential targets for innovative therapies. Modern molecular diagnostics, emerging biomarkers, and point-of-care tools provide opportunities for earlier and more accurate detection, while individualized antimicrobial strategies, surgical interventions, and ART optimization improve patient outcomes. Importantly, the burden of HIV-associated osteomyelitis is disproportionately high in resource-limited settings, underscoring the need for context-adapted approaches. Strategies such as task-shifting, generic antibiotic use, clinical scoring systems, and telemedicine offer practical, scalable solutions to enhance access and quality of care. Future research should prioritize mechanistic insights, evidence-based treatment algorithms, and the validation of resource-adapted models to ensure equitable and effective management globally.

## Data Availability

Not applicable as this a narrative review.

## Research registration unique identifying number (UIN)

Not applicable as this a narrative review.
